# Differential Methylation of *TCF7L2* Promoter in Peripheral Blood DNA in Newly Diagnosed, Drug-Naïve Patients with Type 2 Diabetes

**DOI:** 10.1371/journal.pone.0099310

**Published:** 2014-06-10

**Authors:** Silvia Canivell, Elena G. Ruano, Antoni Sisó-Almirall, Belchin Kostov, Luis González-de Paz, Eduardo Fernandez-Rebollo, Felicia A. Hanzu, Marcelina Párrizas, Anna Novials, Ramon Gomis

**Affiliations:** 1 Department of Endocrinology and Nutrition, Hospital Clinic, Barcelona, Spain; 2 Les Corts Primary Health Care Centre, PHC Research Group, IDIBAPS, Barcelona, Spain; 3 Diabetes and Obesity Laboratory, IDIBAPS, Barcelona, Spain; 4 CIBERDEM, Barcelona, Spain; 5 University of Barcelona, Barcelona, Spain; University of Eastern Finland, Finland

## Abstract

*TCF7L2* is the susceptibility gene for Type 2 diabetes (T2D) with the largest effect on disease risk that has been discovered to date. However, the mechanisms by which *TCF7L2* contributes to the disease remain largely elusive. In addition, epigenetic mechanisms, such as changes in DNA methylation patterns, might have a role in the pathophysiology of T2D. This study aimed to investigate the differences in terms of DNA methylation profile of *TCF7L2* promoter gene between type 2 diabetic patients and age- and Body Mass Index (BMI)- matched controls. We included 93 type 2 diabetic patients that were recently diagnosed for T2D and exclusively on diet (without any pharmacological treatment). DNA was extracted from whole blood and DNA methylation was assessed using the Sequenom EpiTYPER system. Type 2 diabetic patients were more insulin resistant than their matched controls (mean HOMA IR 2.6 vs 1.8 in controls, *P*<0.001) and had a poorer beta-cell function (mean HOMA B 75.7 vs. 113.6 in controls, *P*<0.001). Results showed that 59% of the CpGs analyzed in *TCF7L2* promoter had significant differences between type 2 diabetic patients and matched controls. In addition, fasting glucose, HOMA-B, HOMA-IR, total cholesterol and LDL-cholesterol correlated with methylation in specific CpG sites of *TCF7L2* promoter. After adjustment by age, BMI, gender, physical inactivity, waist circumference, smoking status and diabetes status uniquely fasting glucose, total cholesterol and LDL-cholesterol remained significant. Taken together, newly diagnosed, drug-naïve type 2 diabetic patients display specific epigenetic changes at the *TCF7L2* promoter as compared to age- and BMI-matched controls. Methylation in *TCF7L2* promoter is further correlated with fasting glucose in peripheral blood DNA, which sheds new light on the role of epigenetic regulation of *TCF7L2* in T2D.

## Introduction

Type 2 diabetes (T2D) results from an interaction of genetic risk and environmental factors[1]. The heritability estimates for T2D range from 20% to 80%. The evidence for heritability has been proven with different studies, such as population, family and twin-based studies[2], [3]. Through genome-wide association studies, over 60 loci have been associated with T2D risk[1]. However, the genetic loci discovered to date explain only a small part of the T2D heritability[1]. Reasons for the observed “missing heritability” in T2D include gene-environment interactions, the role of gene variants and epigenetics[1]. Epigenetics refer to heritable changes in gene function that occur without a change in nucleotide sequence. Epigenetic mechanisms could provide a molecular explanation for some unresolved issues in T2D[4], such as discordance within monozygotic twins[5], interindividual variation in age of onset, disease severity and effect of lifestyle factors on T2D risk. Indeed, recent studies propose that specific changes in the epigenome are associated with the onset and progression of diabetes[6], [7], [8], [9]. DNA methylation is the best studied epigenetic modification and influences transcriptional regulation[10]. DNA methylation is a reversible process that can be modulated by both stochastic and environmental stimuli[11]. On the other hand, *TCF7L2* remains the most significant and consistently replicated gene linked to T2D[1], [12]. *TCF7L2* has the strongest effect for T2D (average OR 1.37)[13]) and encodes a transcription factor implicated in wnt signaling and proglucacon transcription [14]. It has been shown that *TCF7L2* expression in human islets was increased 5-fold in T2D and overexpression of *TCF7L2* in human islets reduced glucose-stimulated insulin secretion[15]. However, the precise role of *TCF7L2* with regard to T2D risk is still under investigation. As DNA methylation influences gene expression, we speculated that *TCF7L2* gene could be affected by alterations in DNA methylation in type 2 diabetic patients. Considering that DNA methylation occurs principally in the upstream regulatory regions of the genes[16], we concentrated on the promoter of *TCF7L2* gene. Previous studies have shown that disease-related methylation may be reflected in accessible tissues such as peripheral blood[17].

The aim of this study was to compare the epigenetic profile (defined here as the pattern of DNA methylation on *TCF7L2* promoter in DNA from peripheral blood) between type 2 diabetic patients and age- and BMI-matched controls.

## Materials and Methods

### Ethics Statement

This study was approved by the Clinical research ethical committee of the Hospital Clínic, Barcelona, Spain (25^th^ November 2010, register number 2010/6162) and complies with all laws and international ethics guidelines outlined in the Declaration of Helsinki. All human subjects provided written, informed consent. All samples and clinical data collected were anonymised at source.

### Study design and subjects included

We conducted a case-control study where cases were defined as patients suffering from T2D that were treated only by diet. Cases and controls were recruited from the same primary health center. Eligibility criteria for inclusion of cases and controls were applied as previously cited[18]. Briefly, eligibility criteria for cases were the following: clinical diagnosis of T2D, adequate glycemic control after a period of minimum six months of low-carbohydrate diet and lifestyle interventions, no pharmacological therapy for T2D needed to achieve the glycemic control. In case oral medication was needed for optimal glycemic control, those patients were excluded from the study. Diagnosis of T2D was done following ADA recommendations[19]. Eligibility criteria for controls were as follows: a negative oral glucose tolerance test (OGTT) at recruitment, no previous diagnosis of T2D or prediabetes, no chronic treatment with oral steroids. All controls had an OGTT conducted to confirm they did not have any glucose intolerance. Controls were frequency matched (i.e. match on cell instead of individual[20]) on age and BMI to cases. Physical inactivity was assessed by asking the subjects if they practised at least 30 min of exercise by day. The subjects who answered “no” were classified as “physically inactive”. Subjects addicted to alcoholism or with a history of alcoholism were excluded from the study. Metabolic profile and DNA methylation of *TCF7L2* promoter in peripheral blood DNA profile was studied for all subjects (93 cases and 93 controls).

### Metabolic assessments

All subjects were examined by anthropometric measurements and had fasting metabolic assessments at recruitment. These assessments included fasting serum glucose, fasting serum insulin, glycohemoglobin A1 (HbA1), total cholesterol, triglycerides, high density level (HDL) cholesterol, low density level (LDL) cholesterol, hepatic profile, homeostatic model assessment to quantifiy insulin resistance (HOMA-IR) and homeostatic model assessment to quantifiy beta-cell function (HOMA-B). HOMA-IR was calculated as follows: HOMA-IR  =  (FSI × FSG)/22.5 [21]; HOMA-B  = (20×FSI)/(FSG −3.5), where FSI is the fasting serum insulin concentration (mU/l) and FSG is fasting serum glucose (mmol/l)[22]. All laboratory analyses were performed at the central biochemical laboratory of the Hospital Clinic, Barcelona, Spain.

### DNA methylation analysis

Whole blood samples were stored in the Biobank Hospital Clínic-IDIBAPS; Barcelona, Spain[23]. Genomic DNA was extracted from whole blood for all the subjects studied using standards procedures from the Biobank[23]. Sequenom's MassARRAY platform was used to perform quantitative methylation analysis[24]. This system utilizes MALDI-TOF mass spectrometry in combination with RNA base-specific cleavage (MassCLEAVE). A detectable pattern is then analyzed for methylation status. PCR primers for the amplification of the promoter of *TCF7L2* gene were designed using *Epidesigner* (See Appendix S1). Sequenom's EpiTYPER procedure and protocols include an intern quality control of the methylation data[25]. Bisulfite conversion was done for all samples (all cases and controls) together, with the same reactive preparation, and the same operator. The methylation analysis was done during the same day for all the samples (cases and controls). Methylation data was generated in duplicate for each CpG. There was one run for all cases and another one for all controls, and all were done by the same operator during the same day in the same machine. A fully methylated positive control was included for each run.

### Statistical analysis

Methylation data are generated as β values between 0 and 1, indicating percentage methylation of the original template[26]. Due to the high variability of methylation data over the genomic region analyzed, we decided to do the analysis using each CpG site individually. Descriptive data are presented as the mean and standard deviation (SD) for continuous outcomes, or number and percentage (%) for categorical outcomes. HOMA-IR, HOMA-B, and insulin were compared using non-parametric Mann-Whitney U test because normality and equality of variance could not be assumed. Student's *t* test was used for the comparison of the rest of continuous outcomes and Chi-square test for categorical outcomes. Methylation differences between cases and controls were studied by comparing the methylation means in each CpG site using a non-parametric test (Mann-Whitney U test). Logistic regression models adjusting for age, BMI, gender, waist circumference, smoking status and physical inactivity were built to confirm the unadjusted results. Finally, to study the potential association of methylation data with clinical and biochemical parameters, we did a correlational analysis (calculating Spearman's rank correlation coefficients) and we performed multivariate lineal regression models adjusting for age, BMI, gender, waist circumference, smoking status, physical inactivity and diabetes status for each CpG site. Overall R^2^ values for the models including CpG methylation values, sex, age, BMI, waist circumference, physical inactivity, smoking status and diabetes status are given as percentages. This was done to give an estimate of the association between outcome and methylation. False discovery rate (FDR) correction was used for multiple comparisons[27]. All significance tests were 2-tailed and values of *P*<0.05 were considered significant. All analyses were conducted using the statistical software package Stata version 11 and R Bioconductor.

## Results

### Metabolic profile of the type 2 diabetic patients and controls

Baseline characteristics of the patients included in the study are summarized in Table 1. All patients were overweight (mean BMI of 29.2±3.7 in type 2 diabetic patients vs. mean BMI of 28.8±2.5 in controls, *P* = 0.454). Mean age of all patients was 68 years and there were no significant differences in gender (66.7% were men in the group of cases vs 53.8% in the group of controls, *P* = 0.072). Type 2 diabetic patients had a higher waist circumference as compared to controls (mean waist values of 102.7±9.5 cm vs. 97.9±8.0 cm, *P* = 0.002). Total cholesterol was lower in cases as compared to controls (total cholesterol mean values of 4.8±1.0 mmol/L vs. 5.2±1.1 mmol/L *P* = 0.002). HOMA-IR was higher in cases than in controls (2.6±1.5 vs. 1.8±0.7 in controls, *P*<0.001). HOMA-B was lower in type 2 diabetic patients as compared to controls (75.7±51.1 in type 2 diabetic patients vs 113.6±510.6 in controls, *P*<0.001). Type 2 diabetic patients were less physically inactive as compared to controls (28% vs. 53.8%, respectively *P*<0.001).

**Table 1:** Demographic and clinical characteristics of type 2 diabetic patients and age- and BMI-matched controls.

Variable*	Type 2 diabetic patients (n = 93)	Controls (n = 93)	P Value†
**Demographic characteristics**			
Age, yr	69.1±9.2	66.6±11.7	0.099
BMI, kg/m^2^	29.2±3.7	28.8±2.5	0.454
Waist circumference, cm	102.7±9.5	97.9±8.0	**0.002**
Male sex, (%)	66.7	53.8	0.072
Duration of diabetes, yr	5.4±4.1		
Physical inactivity, %	28.0%	53.8%	**<0.001**
Never smoked, %	50.5%	61.3%	0.261
**Laboratory values**			
Fasting glucose, (mmol/L)	6.4±1.2	4.6±0.3	**<0.001**
Glycated hemoglobin, (%)	5.8±0.6		
Fasting insulin, (pmol/L)	55.6±28.6	52.4±21.0	0.750
HOMA-IR §	2.6±1.5	1.8±0.7	**<0.001**
HOMA-B §§	75.7±51.1	113.6±510.6	**<0.001**
Alanine aminotransferase (ALT), (IU/liter)	13.5±7.9	14.6±7.3	0.486
Aspartate aminotransferase (AST), (IU/liter)	16.6±8.2	19.0±6.0	0.135
Total cholesterol (mmol/L)	4.8±1.0	5.2±1.1	0.002
LDL cholesterol (mmol/L)	2.8±0.8	2.9±0.8	0.782
HDL cholesterol (mmol/L)	1.3±0.3	1.4±0.3	0.262
Triglycerides (mmol/L)	1.4±0.9	1.3±0.8	0.338

* Values shown are means ±SD, unless otherwise indicated.

† P values were calculated with the t test for quantitative variables or Chi-square test for categorical ones, except for HOMA-IR, HOMA-B, fasting insulin, where non-parametric Mann-Whitney U test was applied.

§ HOMA-IR was calculated as [Insulin mlU/l x FSG: (mmol/l)/22.5].

§§ HOMA-B was calculated as (20× FSI)/(FSG −3.5), where FSI is the fasting serum insulin concentration (mU/l) and FSG is fasting serum glucose (mmol/l).

### Quantitative DNA Methylation analysis in peripheral blood of *TCF7L2* promoter in type 2 diabetic patients and controls

Methylation levels in DNA from whole blood of 186 subjects were obtained for 22 sites covering the region between -497 bp and +186 bp according to the ATG position for the *TCF7L2* gene (ENSG00000148737). The heat map showing the methylation values (%) for each CpG site analyzed did not reveal a clearly distinct pattern of methylation between type 2 diabetic patients and controls in the region analyzed (Figure not shown), however some significant differences were actually found. Indeed, multivariate logistic regression models confirmed that 14 out of the 22 CpGs analyzed (64%) showed significant differences in DNA methylation values between type 2 diabetic patients and controls (see adjusted *P*-values in Table 2). When accounting for multiple testing in the multivariate logistic regression models, only 13 out of 22 (59%) remained significant (see adjusted *Q*-values in Table 2). The unadjusted correlational analysis showed that the methylation levels of 16 out of 22 CpG sites (73%) were associated with fasting glucose, 5 out of 22 CpG sites (23%) were associated with HOMA-IR, 9 out of 22 CpG sites (41%) were associated with HOMA-B, 6 out of 22 CpG sites (27%) with total-cholesterol and 2 out of 22 CpG sites (9%) with LDL-cholesterol (see Table 3). After further adjustment, only 4 CpG sites remained significantly correlated with fasting glucose and 1 CpG site with total-cholesterol and LDL-cholesterol (see Table 3). Explained variance of fasting glucose was 62% in CpG 9, CpG 17, CpG 25 and CpG 30, including only adjustment factors. These variances increased to 63%, 66%, 66% and 63%, respectively, after including *TCF7L2* methylation in the model, corresponding to an additional explained variance of 1%, 4%, 4% and 1%, respectively. The variance explained by CpG 27 methylation alone on total cholesterol was up to 5% and up to 4% on LDL-cholesterol.

**Table 2:** Peripheral blood DNA methylation values (in %) for each CpG site analyzed in the *TCF7L2* promoter in type 2 diabetic patients and age- and BMI-matched controls*.

CpG site†	Position (bp) ‡	Type 2 diabetic patients (n = 93)	Controls (n = 93)	P-value	Q-value	Adjusted P-value	Adjusted Q-value
CpG 2	−497	27.8±5.3	31.4±5.6	****<0.001****	**<0.001**	**0.005**	**0.011**
CpG 3	−481	1.6±2.5	1.0±2.3	****<0.001****	**<0.001**	0.313	0.383
CpG 4	−473	1.6±2.5	1.0±2.3	****<0.001****	**<0.001**	0.313	0.383
CpG 5	−466	3.0±1.3	3.1±1.6	0.523	0.524	0.483	0.531
CpG 6	−437	92.8±5.8	90.5±4.8	**0.005**	**0.006**	**0.003**	**0.007**
CpG 7	−434	92.8±5.8	90.5±4.8	**0.005**	**0.006**	**0.003**	**0.007**
CpG 8	−386	89.1±4.3	89.8±5.0	0.345	0.389	0.451	0.522
CpG 9	−382	96.3±2.4	95.2±2.5	**0.002**	**0.003**	**0.035**	0.055
CpG 12	−254	3.0±1.6	7.6±3.3	****<0.001****	**<0.001**	**<0.001**	**0.004**
CpG 14	−214	1.1±1.8	0.7±1.0	0.494	0.524	0.800	0.800
CpG 15	−212	1.1±1.8	0.7±1.0	0.494	0.524	0.800	0.800
CpG 16	−114	36.2±11.2	29.2±16.2	**0.004**	0.006	**0.018**	**0.033**
CpG 17	+5	44.0±7.9	37.3±3.4	****<0.001****	**<0.001**	**<0.001**	**0.004**
CpG 18	+15	97.7±3.6	96.4±2.6	****<0.001****	**<0.001**	0.076	0.105
CpG 19	+18	97.7±3.6	96.4±2.6	****<0.001****	**<0.001**	0.076	0.105
CpG 20	+39	4.4±2.0	5.5±2.7	0.002	**0.003**	**0.002**	**0.006**
CpG 24	+75	52.6±5.6	47.7±6.1	****<0.001****	**<0.001**	**<0.001**	**0.004**
CpG 25	+96	44.0±7.9	37.3±3.4	****<0.001****	**<0.001**	**<0.001**	**0.004**
CpG 26	+107	90.0±4.4	96.4±2.3	****<0.001****	**<0.001**	**<0.001**	**0.004**
CpG 27	+137	14.8±4.3	13.3±4.1	0.065	0.078	**0.020**	**0.034**
CpG 29	+180	90.0±4.4	96.4±2.3	****<0.001****	**<0.001**	**<0.001**	**0.004**
CpG 30	+186	20.9±5.0	17.9±6.0	****<0.001****	**<0.001**	**0.006**	**0.012**

* Values shown are means ±SD.

P values were calculated using the Mann-Whitney U test.

Q values were calculated as estimates of the multiple-testing-corrected false discovery rate (FDR).

Adjusted P-values were calculated by performing a logistic regression analysis adjusting by age, gender, BMI, physical inactivity, smoking status and waist circumference.

Adjusted Q*-*values were calculated as estimates of the multiple-testing-corrected false discovery rate (FDR) on the adjusted P-values.

† CpG dinucleotides have been numbered relative to ATG.

‡ CpG dinucleotide position has been determined according to the ATG position for the *TCF7L2* gene (ENSG00000148737).

**Table 3:** Results of methylation correlation analysis between each CpG site analyzed in the *TCF7L2* promoter and the listed dependent variables*.

CpG site†	Fasting glucose	Fasting insulin	HOMA-IR	HOMA-B	GOT	GPT	Total cholesterol	HDL-cholesterol	LDL-cholesterol	Triglycerides
CpG 2	−0.231 **(0.002)**	−0.112 (0.132)	−0.163 **(0.028**)	0.089 (0.234)	0.003 (0.976)	−0.051 (0.581)	0.101 (0.174)	0.009 (0.902)	0.083 (0.267)	−0.012 (0.871)
CpG 3.4	0.19 **(0.011)**	−0.032 (0.665)	0.046 (0.539)	−0.187 **(0.012)**	−0.041 (0.661)	−0.015 (0.869)	0.017 (0.818)	0.021 (0.783)	0.051 (0.5)	−0.055 (0.461)
CpG 5	−0.021 (0.778)	−0.09 (0.227)	−0.095 (0.203)	−0.015 (0.844)	−0.032 (0.726)	0.003 (0.978)	0.109 (0.141)	0.079 (0.285)	0.064 (0.386)	0.089 (0.232)
CpG 6.7	0.161 **(0.03)**	−0.059 (0.431)	0.022 (0.764)	−0.203 **(0.006)**	−0.055 (0.548)	0.022 (0.808)	−0.07 (0.342)	−0.086 (0.244)	0.017 (0.824)	0.116 (0.116)
CpG 8	−0.116 (0.117)	−0.023 (0.76)	−0.073 (0.328)	0.065 (0.387)	−0.121 (0.183)	0.083 (0.369)	0.019 (0.798)	−0.01 (0.894)	0.001 (0.988)	−0.022 (0.771)
CpG 9	0.218 **(0.003)‡**	0.099 (0.183)	0.174 **(0.019)**	−0.096 (0.195)	−0.059 (0.52)	−0.028 (0.764)	−0.047 (0.528)	−0.046 (0.531)	0.032 (0.663)	0.088 (0.232)
CpG 12	−0.546 **(<0.001)**	−0.063 (0.398)	−0.249 **(0.001)**	0.367 **(<0.001)**	0.176 (0.053)	0.034 (0.716)	0.135 (0.067)	0.105 (0.157)	0.022 (0.763)	−0.129 (0.081)
CpG 14.15	0.044 (0.555)	0.008 (0.913)	0.035 (0.637)	−0.023 (0.759)	0.02 (0.826)	0.006 (0.946)	0.038 (0.613)	0.141 (0.058)	0.002 (0.981)	−0.13 (0.081)
CpG 16	0.108 (0.145)	−0.074 (0.318)	−0.01 (0.889)	−0.107 (0.149)	−0.036 (0.691)	0.014 (0.88)	−0.157 **(0.032)**	0.039 (0.6)	−0.153 **(0.038)**	−0.042 (0.571)
CpG 17	0.283 **(<0.001)‡**	−0.025 (0.733)	0.107 (0.15)	−0.219 **(0.003)**	−0.047 (0.601)	0.032 (0.725)	−0.163 **(0.026)**	−0.024 (0.743)	−0.094 (0.203)	0.037 (0.613)
CpG 18.19	0.213 **(0.004)**	0.013 (0.859)	0.089 (0.229)	−0.137 (0.064)	−0.131 (0.147)	−0.054 (0.558)	−0.117 (0.113)	−0.124 (0.093)	−0.04 (0.59)	0.058 (0.429)
CpG 20	−0.212 **(0.004)**	−0.036 (0.626)	−0.077 (0.298)	0.141 (0.056)	0 (0.997)	−0.063 (0.492)	0.039 (0.599)	0.003 (0.964)	−0.006 (0.933)	−0.008 (0.909)
CpG 24	0.286 **(<0.001)**	−0.074 (0.32)	0.054 (0.465)	−0.275 **(<0.001)**	−0.182 **(0.044)**	−0.092 (0.315)	−0.102 (0.166)	−0.054 (0.463)	−0.044 (0.557)	−0.014 (0.849)
CpG 25	0.283 **(<0.001)‡**	−0.025 (0.733)	0.107 (0.15)	−0.219 (0.003)	−0.047 (0.601)	0.032 (0.725)	−0.163 **(0.026)**	−0.024 (0.743)	−0.094 (0.203)	0.037 (0.613)
CpG 26	−0.504 **(<0.001)**	−0.056 (0.445)	−0.215 **(0.003)**	0.349 **(<0.001)**	0.124 (0.17)	0.076 (0.404)	0.18 **(0.014)**	0.033 (0.651)	0.102 (0.167)	−0.091 (0.215)
CpG 27	0.007 (0.922)	−0.015 (0.837)	0.003 (0.967)	−0.023 (0.759)	0.026 (0.776)	0.13 (0.153)	−0.234 **(0.001)‡**	−0.123 (0.093)	−0.231 **(0.002)‡**	−0.044 (0.555)
CpG 29	−0.504 **(<0.001)**	−0.056 (0.445)	−0.215 **(0.003)**	0.349 **(<0.001)**	0.124 (0.17)	0.076 (0.404)	0.18 **(0.014)**	0.033 (0.651)	0.102 (0.167)	−0.091 (0.215)
CpG 30	0.326 **(<0.001)‡**	−0.03 (0.684)	0.074 (0.316)	−0.24 **(0.001)**	−0.036 (0.687)	−0.001 (0.988)	−0.144 (0.05)	−0.025 (0.734)	−0.066 (0.37)	−0.045 (0.54)

^*^Values shown are Spearman correlation coefficients and p-values in brackets between each CpG site and the dependent variables. In bold are marked the variables that were significant in the unadjusted analysis.

‡ Variables that remained significant after full adjustment by age, gender, BMI, physical inactivity, smoking status, waist circumference and diabetes status in linear regression analyses.

† CpG dinucleotides have been numbered relative to ATG.

## Discussion

In this study, we report the methylation pattern of *TCF7L2* promoter from peripheral blood DNA in drug-naïve type 2 diabetic patients and age- and BMI-matched controls. We found that several CpGs had significant differences between type 2 diabetic patients and controls, although overall the methylation pattern did not show a clear differential pattern related to T2D. These results are consistent with previous data of promoter methylation patterns from peripheral blood DNA where a global directional change in methylation levels that would affect all neighboring CpGs systematically and that would be characteristic of the disease has not been identified[18], [28]. On the other hand, a recent study found some T2D-related methylation patterns in peripheral blood DNA[17] but their analysis did not cover the genomic region we studied. There is great interest to perform methylation profiling in peripheral blood to find methylation disease-related associations since specific methylated regions could be used as potent biomarkers[29]. However, to study how these differentially methylated regions may play a mechanistic role in the development of the disease of interest, the methylation analysis should focus in the tissues relevant for the genes studied. *TCF7L2* is highly expressed in beta-cells, followed by colon, brain, small intestine, monocytes, and lung[30], whereas no expression was detected in lymphocytes T or B. It has been shown that depletion of *TCF7L2* results in reduced GIP-Receptor levels in pancreatic islets and in impaired beta-cell function[31]. In our study, we found that methylation of specific CpG sites on *TCF7L2* promoter in blood was correlated with fasting glucose, total cholesterol and LDL-cholesterol. In line with our results, it has been recently shown that beta-cells cultured with high-glucose-lipid medium presented aberrant DNA methylation in different loci, among which was *TCF7L2* gene promoter[32]. Moreover, Hu et al showed that, while *TCF7L2* promoter was hypermethylated, *TCF7L2* mRNA expression increased, and, unexpectedly, the protein expression of *TCF7L2* was decreased in beta- cells[32]. The mechanisms of this opposite regulation remain unkown, although it could be speculated that DNA methylation may affect the *TCF7L2* splice variants[33], i.e., the increase in mRNA levels could represent transcripts of *TCF7L2* which would encode less active isoforms[32]. Methylation patterns are thought to be tissue-specific[10], [34], [35], thus we might not extrapolate the methylation patterns found in blood to those present in beta-cells. As *TCF7L2* gene is not expressed in blood lymphocytes, we did not perform mRNA expression analyses in peripheral blood. Nevertheless, the first methylome reference in human pancreatic islets has been just published[36]. Dayeh et al performed a genome-wide DNA methylation analysis of human pancreatic islets from type 2 diabetic and non-diabetic donors[36]. In this study, *TCF7L2* gene presented differential methylation values in diabetic pancreatic islets as compared to non-diabetic pancreatic islets. It should be noted though that the region they studied in *TCF7L2* gene is further downstream (3′) than the region we studied.

Type 2 diabetic patients and controls were similar in age and BMI to control for any confounder effect of age and obesity on the results. Moreover, none of the type 2 diabetic patients were on any pharmacological therapy for diabetes. Thus, no confounding effect of antidiabetic drugs or insulin therapy was possible, either. Type 2 diabetic patients received counselling about exercise and healthy diet in order to control their diabetes. This could explain why the % of physically inactive subjects was higher in the control group as compared to the type 2 diabetic patients. The majority of type 2 diabetic patients (67%) were on statins as compared to controls. This could explain the differences in mean total cholesterol between the two groups. Type 2 diabetic patients were in optimal glycemic control (mean glycated hemoglobin 5.8%) and had their clinical diagnosis of T2D recently (mean duration of diabetes was 5 years). Results showed that type 2 diabetic patients were more insulin-resistant than controls, since they presented higher values of HOMA-IR. In concordance to this, type 2 diabetic patients had a higher waist circumference as compared to controls. Higher waist circumference is one component used for the diagnosis of the metabolic syndrome and previous research showed that it correlates with poorer glucose control in type 2 diabetic patients[37]. In contrast, and as expected, beta-cell function was already impaired in type 2 diabetic patients as compared to controls (HOMA-B was significantly lower in type 2 diabetic patients as compared to controls). These data illustrates the fact that impairment of beta-cell function is worse in type 2 diabetic patients as compared to age- and BMI- matched controls. These results are in concordance with the existing literature[38], [39], [40].

The strength of our research is that we have demonstrated that type 2 diabetic patients have differences in concrete CpGs sites of *TCF7L2* promoter as compared to age- and BMI-matched controls. We also found new correlations between fasting glucose, total cholesterol and LDL-cholesterol with DNA methylation in specific CpG sites of *TCF7L2* promoter in DNA from peripheral blood. However, despite accounting for the major confounding factors (age, BMI, diabetes pharmacologic therapy), residual confounding and reverse causation remain possible[41]. As proposed by Relton et al[41], by applying a *“genetical epigenomics”* approach, we could overcome this issue. In our case, the approach would be to study the genetic variants related to the methylation patterns and then to verify whether the correlation with methylation values and fasting glucose and cholesterol remains. However, this was not the goal of the present study.

In conclusion, the targeted epigenetic analysis in DNA from peripheral blood identified differences in specific sites of the *TCF7L2* promoter between type 2 diabetic patients and matched controls. Lipid and glucose blood-parameters were correlated with methylation in specific CpG sites of the *TCF7L2* promoter. Further research should unveil the potential role of these data in the physiopathology of T2D. Our findings add to the growing understanding of the interplay between epigenetics and T2D susceptibility gene *TCF7L2* in the development of the disease.

## Supporting Information

Appendix S1 **Primers used for quantitative DNA methylation analysis.**
(DOCX)
