# Supplementary material for: Differential Methylation of TCF7L2 Promoter in Peripheral Blood DNA in Newly Diagnosed, Drug-Naïve Patients with Type 2 Diabetes
Source: PLoS One. 2014 Jun 10;9(6):e99310. doi: 10.1371/journal.pone.0099310 (PMC4051650; doi:10.1371/journal.pone.0099310)
Supplement: Appendix S1 — Primers used for quantitative DNA methylation analysis. (DOCX) [file pone.0099310.s001.docx]

**Appendix S1. Primers used for quantitative DNA methylation analysis***

| ***TCF7L2* CpGs 1-15 LEFT** | aggaagagagGGTATTTTATTAAGGTAGTGTGTTTTTTT |
| --- | --- |
| ***TCF7L2* CpGs 1-15 RIGHT** | cagtaatacgactcactatagggagaaggctTTTTTCTACTTAAAAATCTTTTTCTCC |
| ***TCF7L2* CpGs 16-31 LEFT** | aggaagagagTTTTTAGGAGAAAAAGATTTTTAAGTAGA |
| ***TCF7L2* CpGs 16-31 RIGHT** | cagtaatacgactcactatagggagaaggctCAAACCCAAAAAACAAATAAAAAAC |

* LEFT: 10-mer tag sequence. RIGHT: T7 promoter tag with an 8 base pair insert (for prevention of abortive cycling and constant 5’ fragment for RNaseA reaction).

Bold case indicate the actual sequence for the primers.

**Methylation analysis.**

Sequenom's MassARRAY platform was used to perform quantitative methylation analysis. This system utilizes MALDI-TOF mass spectrometry in combination with RNA base specific cleavage (MassCLEAVE). A detectable pattern is then analyzed for methylation status. PCR primers for amplification of the promoter of the gene *TCF7L2*  have been designed by using Epidesigner (Sequenom). When it was feasible, amplicons were designed to cover CpG islands in the same region as the 5′ UTR. For each reverse primer, an additional T7 promoter tag for in vivo transcription has been added, as well as a 10-mer tag on the forward primer to adjust for melting-temperature differences. The primers used appear on the above table.

The PCRs have been carried out in a 5 μl format with10 ng/ml bisulfite-treated DNA, 0.2 units of *Taq*DNA polymerase (Sequenom), 1x supplied *Taq* buffer, and 200 mM PCR primers. Amplification for the PCR was done as follows: preactivation of 95°C for 15 min, 45 cycles of 95°C denaturation for 30 s, 56°C annealing for 30 s, and 72°C extension for 30 s, finishing with a 72°C incubation for 4 min. Dephosphorylation of unincorporated dNTPs has been performed by adding 1.7 ml of H_2_O and 0.3 units of shrimp alkaline phosphatase (Sequenom), incubating at 37°C for 40 min, and then for 10 min at 85°C to deactivate the enzyme. The MassCLEAVE biochemistry has been performed as follows: Next, *in vivo* transcription and RNA cleavage was achieved by adding 2 μl of PCR product to 5 μl of transcription/cleavage reaction and incubating at 37°C for 3 h. The transcription/cleavage reaction contains 27 units of T7 RNA&DNA polymerase (Sequenom), 0.64x of T7 R&DNA polymerase buffer, 0.22 μl T Cleavage Mix (Sequenom), 3.14 mM DTT, 3.21 μl H_2_O, and 0.09 mg/ml RNaseA (Sequenom). The reactions have been additionally diluted with 20 ml of H_2_O and conditioned with 6 mg of CLEAN Resin (Sequenom) for optimal mass-spectra analysis.
